# Evaluation of a Resident Wellness Curriculum: A Cross-Sectional Survey in an Internal Medicine Residency Program

**DOI:** 10.7759/cureus.106118

**Published:** 2026-03-30

**Authors:** Ram Prakash Thirugnanasambandam, Shannon Crehan, Pascale G Lafortune, Ben Nguyen, Christine Yun, Harjinder Gill, Patrick Geraghty, Samy I. McFarlane

**Affiliations:** 1 Internal Medicine, State University of New York (SUNY) Downstate Health Sciences University, Brooklyn, USA; 2 General Surgery, St. George's University School of Medicine, St. George's, GRD; 3 Internal Medicine, State University of New York (SUNY) Downstate Health Sciences University/NYC Health and Hospitals, Brooklyn, USA

**Keywords:** american medical association, burnout, internal medicine training, medical residency, wellness curriculum

## Abstract

Introduction

Residency training is a critical period marked by high stress and burnout. Wellness curriculums offer potential solutions, but their effectiveness remains underexplored. This study aimed to assess resident perceptions, awareness, and satisfaction following implementation of a resident-led structured wellness curriculum in an Internal Medicine residency program.

Methods

A cross-sectional survey conducted in May 2024 utilized a 20-point questionnaire distributed to residents across Post-Graduate Year (PGY) levels. The curriculum, based on the American Medical Association's (AMA) six aspects of wellness, was integrated into outpatient rotations. Expert-led talks covered burnout, sleep hygiene, nutrition and fitness, financial planning, emotional health, and fostering relationships. Statistical analysis included Chi-squared tests, with significance set at p < 0.05.

Results

Out of 90 total residents, 48 (53%) participated in the survey. PGY-1 residents constituted 40% of respondents; 58% reported awareness of AMA-defined wellness, and 83% expressed satisfaction with the curriculum. Supportive program leadership and work-life balance were the most valued aspects. 86% recommended continuing the curriculum, suggesting improvements such as increased hospital support staff, upgraded computer systems, mental health workshops, addressing workplace culture, and team outings during didactics. High attendance at wellness talks was observed.

Conclusion

Residents reported high satisfaction with the curriculum and strong support for its continuation. Feedback emphasized the importance of addressing organizational challenges and fostering a supportive culture within the residency program.

## Introduction

Residency training takes approximately three to seven years to complete [1]. However, the training period is crucial for career development and establishing several aspects of their lives, including marriage, raising a family, and having a healthy work-life balance [2]. Sources of stress during this time include poor sleep, financial constraints, a difficult patient population, heavy workload, academic pressure, and social issues [3]. These aspects often add to the overall mental burden of residents and can lead to burnout.

Several studies have looked at the importance of resident wellness along with various related aspects and interventions. Prior studies have reported that most residents reported lower norms of well-being than the general population, with chief stressors being sleep deprivation, extended work hours, and a deficit of personal time [4-6]. Female residents and PGY-1 residents tended to report lower well-being compared to their colleagues [4,7]. Residents reported that their well-being was associated with autonomy, sufficient sleep, regular physical activity, meaningful social connections, and having time for personal activities [8-11]. Three studies focused on exploring interventions to improve resident well-being [12-14]. Having faculty group consultations for family medicine residents showed no measurable impact [12], while having access to the hospital gym was associated with improved well-being outcomes among surgery residents [13]. Anesthesia residents reported reduced anxiety due to the implementation of an evidence-based program that focused on strengthening coping mechanisms [14]. Several studies have reported that regular self-care practices, including adequate sleep, proper nutrition, and exercise, are associated with lower levels of burnout among physicians [15-17]. In addition, psychological health is crucial for physician wellness. In a study by Aaronson et al., only 24% of residents who felt they needed care for their mental health sought treatment, with notable barriers being a lack of time, concerns about confidentiality, and the cost of treatment [18].

Institutional wellness committees, comprising both faculty and residents, are recommended to support the resident well-being and promote systemic initiatives to improve the clinical learning environment [19,20]. Programs may combine passive strategies, such as lectures/ resources, and active approaches such as workshops/ outings to address the challenges (stress, behavioral issues, financial problems, and mental health concerns) faced by residents [21]. A multi-component approach covering resilience, professionalism, emotional and social wellness, financial literacy, team building, and mindfulness has been shown to have significant benefits [22-24]. Additionally, focusing on managing ethical and interpersonal challenges with the addition of retreats, workshops, and social outings can be used to supplement wellness [25-27]. Studies on implemented curriculums have demonstrated improved physician wellness and reduction in burnout [28,29].

Our program therefore designed and implemented a wellness curriculum based on various recommendations to help our Internal Medicine (IM) residents learn more about the various aspects of wellness and how best to implement measures in their daily clinical and personal environment.

## Materials and methods

Study design and population

This study employed a cross-sectional observational design, utilizing a questionnaire distributed to all residents in an Internal Medicine program that had implemented a wellness curriculum. The curriculum was based on the American Medical Association’s (AMA) definition of wellness, which includes six key aspects intended to support resident well-being among physician trainees: nutrition, fitness, emotional health, preventive care, financial health, and mindset adaptability [30]. The questionnaire was distributed via email, and participation was entirely voluntary.

Implementation

The wellness curriculum was designed in alignment with the AMA’s six key aspects of wellness: nutrition, fitness, emotional health, preventive care, financial health, and mindset adaptability. Based on these recommendations, expert speakers from each domain were identified and invited to give talks to the residents. These talks were structured to provide residents with actionable strategies for incorporating wellness into their daily routines and professional lives.

The residency program follows an X+Y schedule, with "X" representing four weeks of inpatient rotations and "Y" representing two weeks of outpatient care. The wellness curriculum was implemented during the outpatient blocks (Y weeks) to ensure that residents had more dedicated time for didactics and, therefore, could participate more effectively in the wellness sessions.

Inclusion criteria

All 90 residents across Post-Graduate Year (PGY) levels who were active during the academic year 2023-2024 were included in the study.

Data collection

A 20-point questionnaire was emailed to all residents after the conclusion of the wellness curriculum in May 2024. Demographic information was collected based on the residents’ PGY level and the firm they belonged to within the internal medicine residency. The questionnaire was developed internally based on the American Medical Association’s six wellness domains and was intended for program evaluation purposes. The survey instrument was not previously validated. The questionnaire was created using SurveyMonkey software and distributed via institutional emails.

Statistical analysis

As this was a cross-sectional exploratory study, the sample size was not determined. All residents who attended the curriculum were asked to complete the survey. Sample size was based on practical feasibility and preliminary pilot data, rather than a formal power analysis. The majority of the data are presented as counts (N) and percentages (%). Responses to multiple-choice questions were summarized in tables for a clear and concise presentation of the findings. A Chi-squared test was used to compare responses across demographic segments. The threshold for statistical significance was set at p < 0.05. All statistical analyses were conducted using GraphPad Prism Software (Version 5.0h; GraphPad Software, San Diego, CA).

## Results

A total of 48 (53%) out of 90 residents across all PGY levels and firms responded (Table 1). PGY-1 residents represented the largest group, comprising 19 residents (40%), followed by 15 PGY-2 residents (31%) and 14 PGY-3 residents (29%). In terms of firm distribution, Firm B accounted for the highest proportion of respondents with 20 residents (42%), while Firm A and Firm C had 15 (31%) and 13 (27%) participants, respectively.

**Table 1:** Distribution of residents classified by firm PGY, Post-Graduate Year. A chi-squared test was performed to test PGY variability within each firm. p-values are shown

Firm	PGY1 N (%)	PGY2 N (%)	PGY3 N (%)	Total N (%)	p-value
Firm A	6	5	4	15 (31%)	0.74
Firm B	8	6	6	20 (42%)	0.74
Firm C	5	4	4	13 (27%)	0.99
Total	19 (40%)	15 (31%)	14 (29%)	48 (100%)	0.52

**Figure 1:**
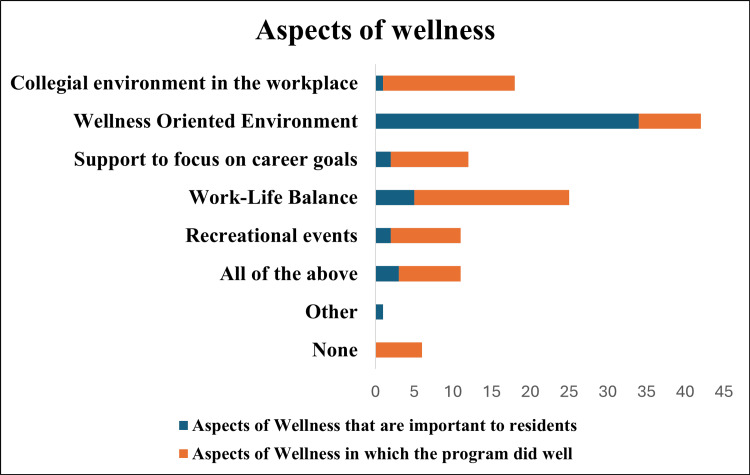
Aspects of wellness

Residents were asked about the aspects of wellness (Figure 1) that they considered important and how they felt the program performed in addressing these areas. The most valued aspect was having the presence of institutional structures that facilitate wellness programs, with 34 residents identifying it as a priority. Other aspects included maintaining a good work-life balance (5), focusing on career goals (2), regular resident recreational activities (2), and fostering a collegial environment (1). Three respondents chose all the options. In terms of program performance, 20 residents reported that the program excelled in promoting work-life balance, followed by 17 who reported that it fostered a collegial environment.

Of the respondents, 28 (58%) were aware of the AMA’s definition of wellness, while 20 (42%) were not (Table 2). Regarding the program's implementation of the wellness curriculum, 40 (83%) residents expressed satisfaction with its execution during outpatient blocks. Two residents indicated dissatisfaction, four suggested incorporating the curriculum during inpatient blocks, and two suggested incorporating it during both inpatient and outpatient blocks. Attendance rates for the wellness talks are summarized (Table 3).

**Table 2:** Awareness and implementation of the wellness curriculum AMA, American Medical Association; Y, Outpatient blocks

Response	Awareness of wellness defined by AMA N (%)	Implementation of the wellness curriculum by the program in the Y block N (%)
Yes	28 (58%)	40 (83%)
No	20 (42%)	2 (4%)
Inpatient Block	NA	4 (8%)
Inpatient + Outpatient Block	NA	2 (4%)
Total	48 (100%)	48 (100%)

**Table 3:** Resident attendance of wellness talks

Wellness topic	Attended talk N (%)	Attended but did not find it useful N (%)	Did not attend N (%)	Did not answer N (%)
Resident Burnout	30 (62%)	6 (20%)	12 (25%)	6 (12%)
Sleep Hygiene	36 (75%)	5 (14%)	6 (13%)	6 (12%)
Importance of Nutrition	33 (69%)	5 (15%)	9 (19%)	6 (12%)
Financial Planning	33 (69%)	6 (18%)	8 (16%)	7 (14%)
Emotional Health and Fostering Relationships	32 (67%)	6 (19%)	8 (16%)	8 (16%)

Several respondents offered suggestions for enhancing the talks, reflecting active engagement and interest in improving the curriculum (Table 4). The majority of respondents, 41 (86%), supported the continuation of the wellness talks as a regular component of the program (Table 5).

**Table 4:** Resident feedback on further improvement to implemented talks

Topics of Wellness	Had suggestions N (%)	Did not have suggestions N (%)
Resident Burnout	18 (37%)	30 (63%)
Sleep Hygiene	16 (33%)	32 (67%)
Importance of Nutrition	15 (31%)	33 (69%)
Financial Planning	13 (27%)	35 (73%)
Emotional Health & Fostering Relationships	13 (27%)	35 (73%)

**Table 5:** Resident perception on implementing resident wellness in the program

Response	Implementation of the wellness curriculum by the program in the future N (%)
Yes	41 (86%)
No	3 (6%)
Other	2 (4%)
Did not answer	2 (4%)
Total	48 (100%)

## Discussion

This study provides valuable insights into resident wellness and engagement with the program's initiatives. Residents highlighted the need for institutional structures that facilitate wellness programs and work-life balance as key priorities, reflecting the importance of a nurturing and collegial environment. The wellness curriculum, implemented during outpatient blocks, was associated with high levels of reported satisfaction, with residents actively participating in the talks and offering suggestions for improvement. The strong support for continuing these sessions suggests that residents viewed them as relevant within the context of residency training.

Multiple studies have explored burnout among resident physicians [31-33]. The study by Rosen et al. showed that PGY-1 residents at an IM residency program showed an increased prevalence of sleep deprivation (9% to 43% with p=0.0001), moderate depression (4.3% to 29.8% with p=0.0002), and reported burnout (4.3% to 55.3% with p<0.0001) from baseline to the end of their intern year [31]. Another study done among 115 IM residents reported that 76% were found to meet burnout criteria as established by the Maslach Burnout Inventory (MBI) and that burnout was associated with self-reported suboptimal patient care practices (56% vs 21% with p=0.004) [32]. The study by Martini et al. also reported that 63% of IM residents also reported burnout in a comparison study of burnout among different specialties [33].

Despite different studies showing that IM residents frequently report various aspects of burnout, the current literature does not report any major targeted interventions by residency programs to help educate and alleviate the burden faced by residents. On a system level, it is important to emphasize organizational and individual-based interventions. Reducing work hours and having strict caps on patient load and on-call periods have mostly shown mixed success [34,35]. Psychological interventions should also be considered as potential strategies for supporting wellness, although this area remains under-researched. This study assessed self-reported perceptions following the implementation of a wellness curriculum that focused on educating IM residents on various aspects of wellness, with the hope of promoting self-motivated implementation of wellness among our IM residents.

Our residency program ensures an equal distribution of residents across PGY levels and firms (A, B, and C) within the X+Y block system, where 'X' represents the inpatient block and 'Y' the outpatient block. Each block spans two weeks, ensuring that all firms follow a consistent schedule of two inpatient blocks followed by one outpatient block. Our results highlight a high level of participation among PGY-1 residents, who comprised 19 (40%) of respondents. Statistical analysis showed no significant differences in PGY distribution among the firms (p = 0.741-0.992), indicating an even distribution of respondents across firms and PGY levels. In a study of 109 EM residents, PGY-1 residents reported significantly lower lifestyle satisfaction compared to PGY-2 and PGY-3 residents (mean ratings: 1.29, 1.66, and 1.70, respectively; p < 0.001). However, there were no significant differences between PGYs in stress ratings for work relationships, work environment, or patient responsibilities [7]. These findings suggest that wellness initiatives may be relevant across all PGY levels, as work-related stressors are reported throughout training.

The questionnaire first asked residents to identify the aspects of wellness they felt were most important to gauge their baseline expectations in an Internal Medicine residency program. This was followed by a question on which aspects of wellness they thought their program excelled at addressing. Respondents had the choice to choose multiple options across these two questions. Having institutional structures that facilitate wellness programs was identified as the most important aspect of wellness by residents across all PGY levels (34 (71%) respondents). These findings suggest alignment between resident priorities and structured components of the curriculum. Conversely, the program performed well in promoting a work-life balance, with 20 (42%) respondents recognizing it as a strength. Interestingly, only five residents had initially identified work-life balance as a priority, which may reflect differences between perceived priorities and reported program strengths. The study by Msheik-El Khoury et al. found that strong program director-resident relationships significantly improved residents' well-being and reduced suboptimal patient care practices through dimensions of burnout and engagement (p < 0.05) [36]. These findings provide context for the role of leadership and institutional support in resident wellness. Approximately 28 (58%) of respondents acknowledged that they were aware of the various aspects of wellness, and 40 (83%) were happy with its implementation during the outpatient blocks. 

The next couple of questions focused on identifying the attendance rates of residents on our curated wellness talk based on the AMA’s definition of wellness [30] as seen in Table 4. The Sleep Hygiene talk had the highest attendance, with 36 (75%) residents participating. This was followed by talks on Nutrition and Financial Planning, each attended by 33 (69%) residents, Emotional Health and Fostering Relationships with 32 (67%) attendees, and Burnout with 30 (62%) attendees. While attendance rates provide descriptive information regarding participation, responses also demonstrated varying levels of perceived utility. For instance, six (20%) attendees for the Burnout session and five (14%) for the Sleep Hygiene session did not find the content useful. These findings indicate variability in perceived usefulness across topics. Additionally, the focus of the talks, addressing stress, behavioral challenges, financial literacy, and mental health concerns, aligns with evidence from previous studies highlighting these as common residency challenges [19,22,37]. Attendance rates for talks held as part of the weekly didactics session were documented as part of the structured curriculum.

The topic of Burnout resulted in the highest resident feedback, with 18 (37%) respondents being enthusiastic about giving suggestions, followed by 16 (33%) respondents for the talk on sleep hygiene, 15 (31%) respondents for the talk on Nutrition, and 13 (27%) respondents for the talks on financial wellness and the final talk on emotional health and importance of fostering relationships. These numbers indicate the distribution of written feedback across topics. Residents provided several suggestions for improving the wellness curriculum, emphasizing the need for organizational-level changes. Common themes included increasing hospital support staff and upgrading computer systems to facilitate the timely completion of clinical responsibilities, thereby allowing more time for personal activities and self-care. Some respondents noted difficulties in implementing wellness strategies, citing limited time to prepare meals and challenges in following recommendations during residency. Others highlighted a need for addressing workplace culture, referring to difficult encounters with attendings and residents from other departments and a lack of visible support for addressing these issues, particularly within the inpatient service. Suggestions for future enhancements included incorporating mental health workshops or team outings during didactic time to foster a more supportive environment.

In response to the question about continuation, most respondents (86%) expressed strong support for maintaining the curriculum, reflecting its positive impact. This finding indicates that the majority of respondents viewed the wellness initiative favorably within the context of the program.

The study’s limitations include its cross-sectional design as well as its reliance on self-reported data, as they may introduce selection and recall bias, as participation in the study was voluntary, and characteristics of non-responders were not collected or compared to those of respondents. The small sample size and focus on a single residency program also limit the generalizability of the findings. Importantly, the study did not include a pre-post comparison, control group, or baseline assessment of wellness, and therefore cannot assess changes over time or causal impact. Additionally, the survey instrument was internally developed and not a previously validated tool, and outcomes were limited to subjective perception of awareness and satisfaction rather than objective or validated measures such as the Maslach Burnout Inventory. The study also does not evaluate longitudinal effects or measurable reductions in burnout or psychological distress. As this is an exploratory study, no formal power calculation was conducted. Therefore, while this study offers valuable insights into an internal medicine wellness curriculum, the limited sample size restricts the generalizability of our findings to broader populations. Further studies may address these limitations by considering broader, longitudinal designs to assess long-term effects, looking at implementation across multiple residency programs and specialties, and incorporating qualitative feedback and objective metrics.

## Conclusions

This study describes resident perceptions of a structured wellness curriculum based on the AMA’s six dimensions of wellness on resident engagement and satisfaction, with 86% supporting its continuation. High attendance rates and active feedback on wellness talks provide descriptive information regarding participation and reported satisfaction. The principal areas that residents highlighted as critical for their wellness were the involvement of program leadership and an open discussion on any organizational barriers. These findings suggest that structured wellness initiatives may be acceptable within residency training programs. Further studies incorporating longitudinal and validated outcome measures are needed to assess measurable effects of burnout and various aspects of resident well-being.

## Figures and Tables

**Figure 2:**
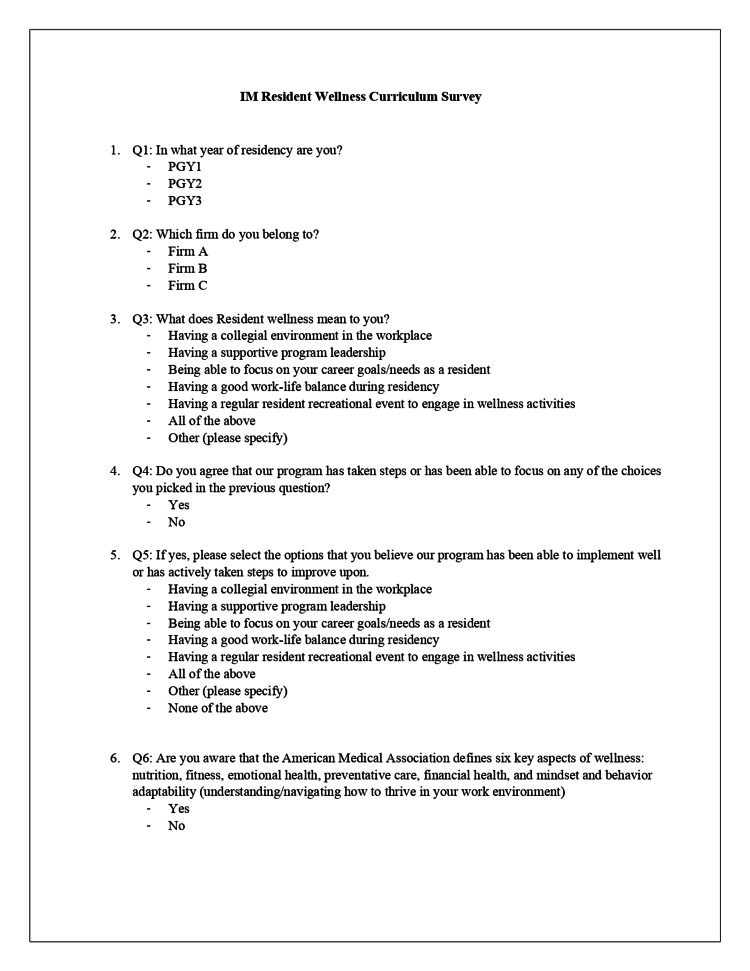
Survey page 1

**Figure 3:**
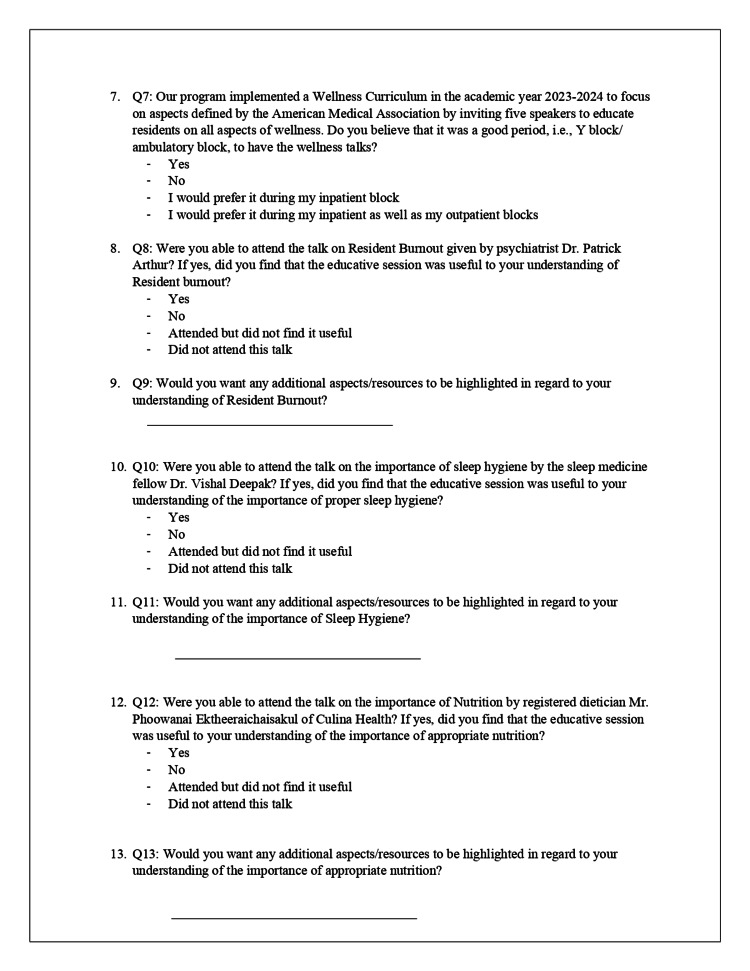
Survey page 2

**Figure 4:**
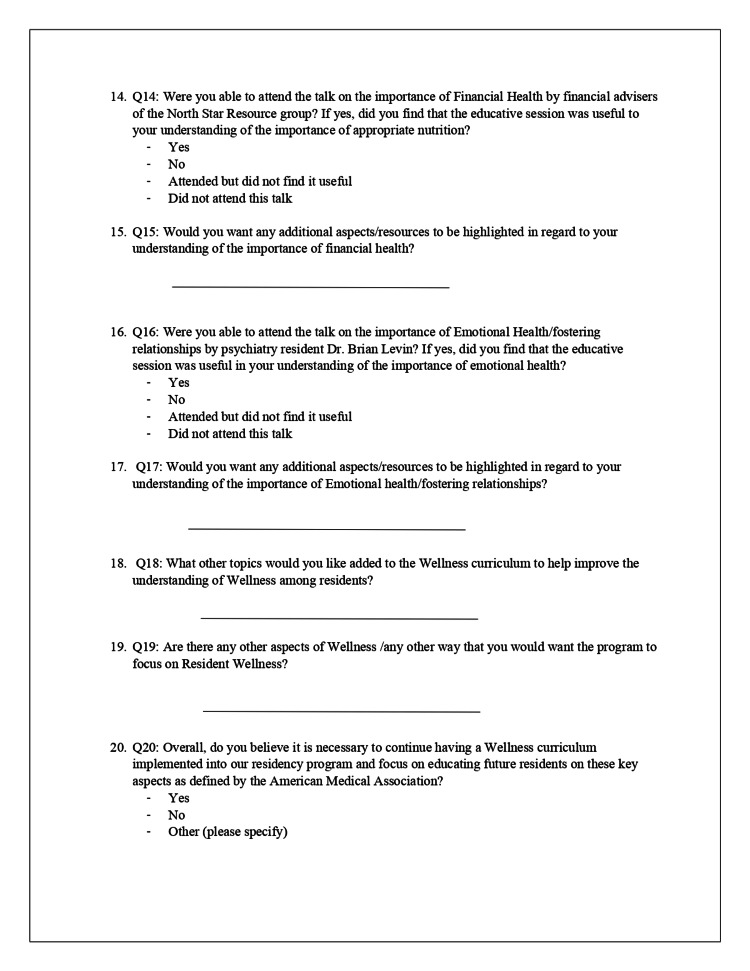
Survey page 3
